# Deciphering miRNA transcription factor feed-forward loops to identify drug repurposing candidates for cystic fibrosis

**DOI:** 10.1186/s13073-014-0094-2

**Published:** 2014-12-02

**Authors:** Zhichao Liu, Jürgen Borlak, Weida Tong

**Affiliations:** Division of Bioinformatics and Biostatistics, National Center for Toxicological Research, U.S. Food and Drug Administration, 3900 NCTR Road, Jefferson, AR 72079 USA; Centre for Pharmacology and Toxicology, Hannover Medical School, Carl-Neuberg-Straße 1, 30625 Hannover, Germany

## Abstract

**Background:**

Cystic fibrosis (CF) is a fatal genetic disorder caused by mutations in the CF transmembrane conductance regulator (CFTR) gene that primarily affects the lungs and the digestive system, and the current drug treatment is mainly able to alleviate symptoms. To improve disease management for CF, we considered the repurposing of approved drugs and hypothesized that specific microRNA (miRNA) transcription factors (TF) gene networks can be used to generate feed-forward loops (FFLs), thus providing treatment opportunities on the basis of disease specific FFLs.

**Methods:**

Comprehensive database searches revealed significantly enriched TFs and miRNAs in CF and CFTR gene networks. The target genes were validated using ChIPBase and by employing a consensus approach of diverse algorithms to predict miRNA gene targets. STRING analysis confirmed protein-protein interactions (PPIs) among network partners and motif searches defined composite FFLs. Using information extracted from SM2miR and Pharmaco-miR, an *in silico* drug repurposing pipeline was established based on the regulation of miRNA/TFs in CF/CFTR networks.

**Results:**

In human airway epithelium, a total of 15 composite FFLs were constructed based on CFTR specific miRNA/TF gene networks. Importantly, nine of them were confirmed in patient samples and CF epithelial cells lines, and STRING PPI analysis provided evidence that the targets interacted with each other. Functional analysis revealed that ubiquitin-mediated proteolysis and protein processing in the endoplasmic reticulum dominate the composite FFLs, whose major functions are folding, sorting, and degradation. Given that the mutated CFTR gene disrupts the function of the chloride channel, the constructed FFLs address mechanistic aspects of the disease and, among 48 repurposing drug candidates, 26 were confirmed with literature reports and/or existing clinical trials relevant to the treatment of CF patients.

**Conclusion:**

The construction of FFLs identified promising drug repurposing candidates for CF and the developed strategy may be applied to other diseases as well.

**Electronic supplementary material:**

The online version of this article (doi:10.1186/s13073-014-0094-2) contains supplementary material, which is available to authorized users.

## Background

Cystic fibrosis (CF) is a lethal autosomal recessive disorder that mostly affects Caucasians with approximately 30,000 cases in the United States and about 70,000 cases reported worldwide. It is caused by mutations in the CF transmembrane conductance regulator (CFTR) gene [1] which codes for an ion channel to regulate the balance between the transport of chloride and the movement of water through an epithelial barrier. Mutations in the CFTR results in altered mucus and thickened secretions to promote chronic infection and inflammation [1]. Note that the mutations are grouped into different classes either affecting the quantity or function or a combination of both of the CFTR protein. Although the molecular causes for CF are well understood and >1,000 mutations have been identified, the treatment of CF is complex and mostly relies on the use of antibiotics. Currently, there is no cure for CF and drug treatment can only ease symptoms by influencing mucus production and the restoration of pulmonary surfactant, the prevention of inflammation and infection, and by the combined use of nutritional supplements [2]. Despite some advances in the treatment and management of disease, the median age of survival for CF patients is still only about 40 years [2].

In 2012, the US Food and Drug Administration (FDA) approved Kalydeco (ivacaftor) for its use in CF patients. This drug modulates CFTR activity and fulfilled a promise made more than 20 years ago when a mutated CFTR was first discovered and researchers spoke optimistically about developing drugs to restore the function of the mutated protein [3]. The successful development of Kalydeco is a milestone in the treatment of CF patients; however whether patients will be able to afford the drug is unclear, making its widespread adoption and use questionable. In the UK, regulators only agreed to approve Kalydeco after Vertex Pharmaceuticals reduced its official list price to £182,625 ($297,000) per year per patient [4] and the drug is intended for use in CF patients of the G551D genotype only and must be aged 6 years and above.

Importantly, to address unmet needs in rare and neglected diseases, drug repurposing of approved drugs has been advocated and attracted significant attention from academia, pharmaceutical industry and governmental agencies [5,6] and included the use of statins (e.g., simvastatin) for the treatment of adult CF [7]. Apart from its lipid-lowering effects, statins influence the production of pro-inflammatory cytokines and chemokines. Moreover, statins modulate nitric oxide (NO) production by inhibiting the RhoGTPase pathway, thereby improving NO and inflammatory components in pathogen infected lungs of CF patients [8], as evidenced in clinical studies [9,10].

An identification of drug-repurposing candidates for CF based on a systematic analysis of an entire drug landscape has not been attempted. We therefore explored a computational strategy based on the drug-repurposing principle that integrates diverse data, including data from emerging molecular technologies such as expression of microRNA (miRNA), and transcription factors (TFs) to promote the rational use of market drugs for the treatment of CF.

For this purpose, feed-forward loops (FFL) were constructed and FFLs are defined as regulatory network motifs, whose connectivity patterns occur much more frequently than randomized in ‘control’ networks [11]. A FFL usually consists of two regulatory elements, one of which controls the other to regulate gene expression together [11]. FFLs have been demonstrated to play important roles in disease development and contributed to an understanding of underlying mechanism [12]. For instance, the two regulatory elements can be defined as two TFs or one TF plus one miRNA. Taylor *et al.* [13] detected a nuclear factor, erythroid 2-related factor (Nrf2), that regulated a FFL which was involved in the protective response to oxidative stress in a mouse disease model. Hall *et al.* [12] reported a type I interferon (IFN) FFL in the pathogenesis of autoimmune rheumatic diseases. Guo *et al.* [14] identified 32 schizophrenia specific FFLs consisting of miRNA, TF, and genes. Afshar *et al.* [15] explored FFLs entailing miRNAs, TFs, and genes in prostate cancer. These proof of concept studies encourage the development of disease specific FFL that can be applied to the process of drug repurposing. Here we hypothesized the existence of a set of FFLs in CF where the two regulatory elements are defined by specific TFs and miRNAs, respectively.

Notably, miRNAs are 18 to 25 nt long non-coding RNAs that function in the transcriptional and post-transcriptional regulation of gene expression [16]. miRNAs are involved in different biological processes such as differentiation, apoptosis, and stress response [17], and miRNAs can interact with the 3′UTR of target mRNAs via base-pairing to facilitate the recruitment of a ribonucleoprotein complex that either blocks cap-dependent translation or triggers target mRNA deadenylation and degradation [17]. An increasing number of miRNAs have been identified to regulate cancers [18,19], multiple sclerosis [20], diabetes [21], hepatotoxicity [22], and cardiovascular diseases [23]. miRNAs have also been reported to play a crucial post-transcriptional role in CF [24-28]. For example, miR-126 was shown to regulate the inflammatory signaling pathway and was reported to be decreased in CF respiratory epithelium as compared to non-CF bronchial epithelial cells *in vivo* and *in vitro* [29]. Likewise, TFs are key regulators in the control of gene expression by translating *cis*-regulatory codes [30]. Due to their function and regulatory logic [31], miRNA and TFs co-regulate the same genes in a complex manner and are therefore suitable elements to construct FFLs.

We therefore hypothesized the existence of a set of FFLs which are composed of both TFs and miRNA to regulate genes in CF and CFTR. Consequently, we constructed CF and CFTR-specific FFLs, and studied the effects of market drugs by inferring perturbations of disease-specific FFLs with the aim to determine their potential utility in the treatment of CF. We focused on approved drugs without boxed warning and are considered to be safe at affordable prices. As a result, we identified market drugs as putative candidates for CF treatment. Strikingly, out of the 48 repurposing drug candidates 26 were confirmed with literature reports and/or existing clinical trials relevant to the treatment of CF patients thus providing evidence for the utility of the employed approach.

## Material and methods

### CF and CFTR associated gene regulations

Initially, we collected information from diverse public repositories including the Genetic Association Database (GAD) [32,33], Orphanet [34,35], the Online Mendelian Inheritance in Man (OMIM) [36,37], the Function disease ontology annotation (FunDO) [38,39], and PubMed reports (see also Table 1). The broad and diverse information was validated by different experimental platforms. Eventually, a comprehensive list of differentially expressed genes (DEG) was compiled using diverse data sets from cystic fibrosis patients with mild and severe lung disease based on tissue samples obtained from bronchial brushings or nasal epithelium as well as rectal epithelia of CF and non-CF individuals (GEO submission GSE2395, GSE55146, and GSE15568). Collectively, a total of 1,042 DEGs were compiled (Additional file 1: Table S1). To discern CFTR-associated gene regulations from CF-related DEGs the data reported by Ramachandran *et al.* [25] were considered and included 419 unique genes (Additional file 1: Table S1).

**Table 1:** **Summary of genes and miRNAs used in this study**

**CF-related miRNAs/genes**		**CFTR-related miRNAs/genes**	
**miRNAs**		**miRNAs**	
HMDD	1	TLDA experiment	113
miR2Disease	0	**Genes**	
PhenomiR	0	Literatures	391
Literatures	7		
Bhattacharyya *et al.* data	22		
Oglesby *et al.*	93		
**Genes**			
GAD	19		
Orphanet	3		
OMIM	4		
Disease Ontology	49		
Nasal respiratory epithelial (GSE2395)	565		
Human bronchial epithelium (GSE55146)	393		
Human epithelial cells (GSE15568)	96		

### MiRNA networks of CF-regulated genes (miRNA → gene/TF)

To identify CF-specific miRNAs, data from different sources were integrated, including a literature search using the keywords ‘miRNA’ and ‘cystic fibrosis’ in PubMed. Here, we focused on miRNA expression profiling studies in CF patient samples and considered particularly the findings of Oglesby *et al.* [40] and Bhattacharyya *et al.* [41] which had information on 93 and 22 regulated miRNAs, respectively.

Furthermore, to be able to distinguish between CF and CFTR miRNAs networks and to identify commonly regulated ones, data obtained from well-differentiated primary human airway epithelial cultures were considered as reported in Ramachandran *et al.* [25]. There were 112 CFTR associated miRNAs of note (Additional file 2: Table S2).

MiRNA analysis and target prediction was done with the TargetScan algorithm [42] and included the search for the presence of conserved 8mer or 7mer sites that match the seed region of the miRNA. The functional annotation of predicted targets is based on experimental validation [43,44] and in the case of miRNA → gene/TF pairs to be considered conserved in *homo sapiens* a total context score higher than -0.4 was applied [45]. To confirm miRNA targets in CF and CFTR networks and to distinguish among individual TFs involved, the predicted target genes were mapped onto a human TFs list in the ChIPBase [46].

### Transcription factor networks of CF regulated genes (TF → gene/miRNA)

The TF and gene/miRNA relationship data were extracted from the ChIPBase [46]. ChIPBase aims to provide high confident information on the transcriptional regulation of long non-coding RNA and miRNA genes from ChIP-Seq data. The data were curated from sources such as the NCBI GEO database [47], ENCODE [48], the modENCODE databases [49,50], and PubMed literature citations. Thus, the TFs related to CF and CFTR gene/miRNA networks were extracted from the human hg19 organism with regulatory regions (upstream: 5 kb; downstream: 1 kb).

### CF protein-protein interaction network

**Figure 1:**
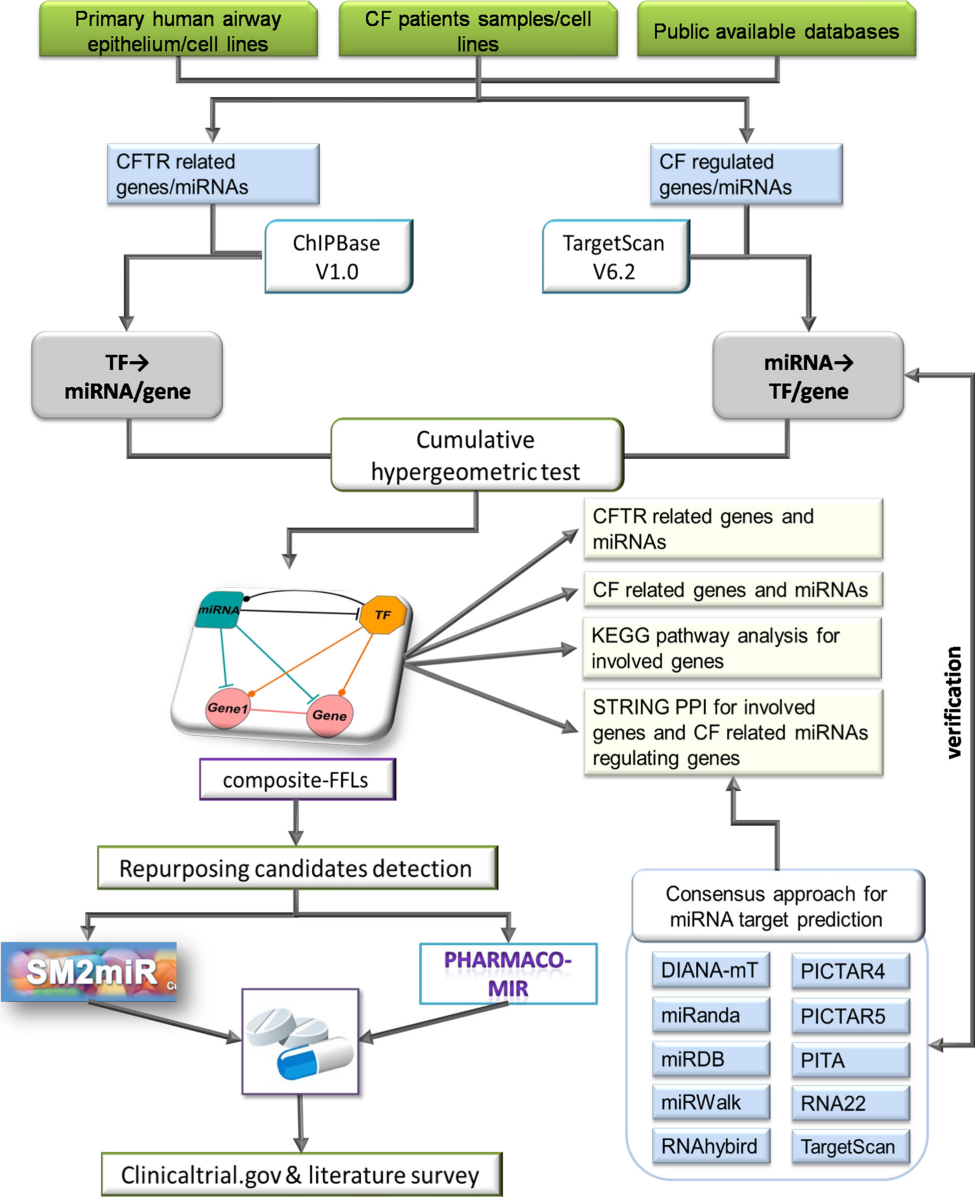
**An outline of the work flow.**

The STRING 9.1 version [51] was applied to study protein-protein-interaction (PPI) using input data derived from [27] and CF patient samples (GEO submission GSE2395, GSE55146, and GSE15568). Initially, a total of 123 CFTR-associated genes were considered and based on 80 genes that are part of the 15 constructed FFLs. A total of 135 PPIs were observed. Furthermore, for nine disease-specific FFLs and the 66 genes associated with it, a total of 97 PPIs were observed. Additionally, for nine out of 15 FFLs the disease-specific regulation of miRNA was validated by a consensus approach by employing 10 different algorithms, that is, DIANA-microT [52], miRanda [53], miRDB [54], miRWalk [55], RNAhybrid [56], PICTAR4 [57], PICTAR5 [57], PITA [58], RNA22 [59], and TargetScan [60] (see Figure 1). Gene targets were considered positive only when confirmed by at least eight algorithms. Importantly, the STRING analysis provided high confidence PPI interactions based on the neighborhood, gene fusion, co-occurrence, co-expression, experiments, text-mining, and so on. In this study, only interactions with confidence scores higher than 0.4 were extracted.

### Enrichment of significantly regulated miRNA and TF in CF gene networks

To assess the statistical significance for genes that were co-regulated by both miRNA and TF, the cumulative hypergeometric test was employed based on the common CF and CFTR-specific genes of any pair of miRNA and TF as described by the following formula [45]:

$$ \raisebox{1ex}{$\mathrm{p}=1-{\Sigma}_0^{\left|{\mathrm{N}}_{\mathrm{miR}}\right|{\displaystyle \cap }{\left|{\mathrm{N}}_{\mathrm{TF}}\right|}^{\left.\left\{\left(\begin{array}{c}\hfill \left|{\mathrm{N}}_{\mathrm{miR}}\right|\hfill \\ {}\hfill \mathrm{i}\hfill \end{array}\right)\right.\left(\begin{array}{c}\hfill \mathrm{Total}-\left|{\mathrm{N}}_{\mathrm{miR}}\right|\hfill \\ {}\hfill \left|{\mathrm{N}}_{\mathrm{TF}}\right|-\mathrm{i}\hfill \end{array}\right)\right\}}}$}\!\left/ \!\raisebox{-1ex}{${}^{\left(\begin{array}{c}\hfill \mathrm{Total}\hfill \\ {}\hfill \left|{\mathrm{N}}_{\mathrm{TF}}\right|\hfill \end{array}\right)}$}\right., $$

where *N*_*miR*_ denotes the number of target genes for a given miRNA, *N*_*TF*_ represents the number of target genes for the corresponding TF, and *Total* is the number of common genes between all the CF- and CFTR-related genes regulated by TFs and repressed by miRNAs. The Benjamini-Hochberg multiple testing corrections were used to adjust the *P* values (function *mafdr.m* from MATLAB 7.10.0 (R2010a)), and only those pairs with justified *P* values less than 0.05 were considered.

### Drug effects on miRNA expression

The effects of drugs on individual miRNAs were compiled from SM2miR [61]. In the present study, only FDA-approved drugs were considered to be potential repurposing candidates for CF. Moreover, the miRNA-gene-drug relationship was extracted from PharmacomiR [62] that provides miRNA pharmacogenomics data manually curated from literatures.

## Results

### CF- and CFTR-related gene and miRNA expression changes

An outline of the work flow is given in Figure 1, and a summary of CF- and CFTR-related gene and miRNA data are given in Table 1. Initially, a comprehensive list of differentially expressed genes (DEG) was compiled using diverse data sets from CF patients in addition to literature findings regarding CFTR-associated gene networks. Subsequently, common regulations of DEGs by TFs and miRNAs were investigated by means of databases searches in addition to experimental data retrieved from literature searches.

**Figure 2:**
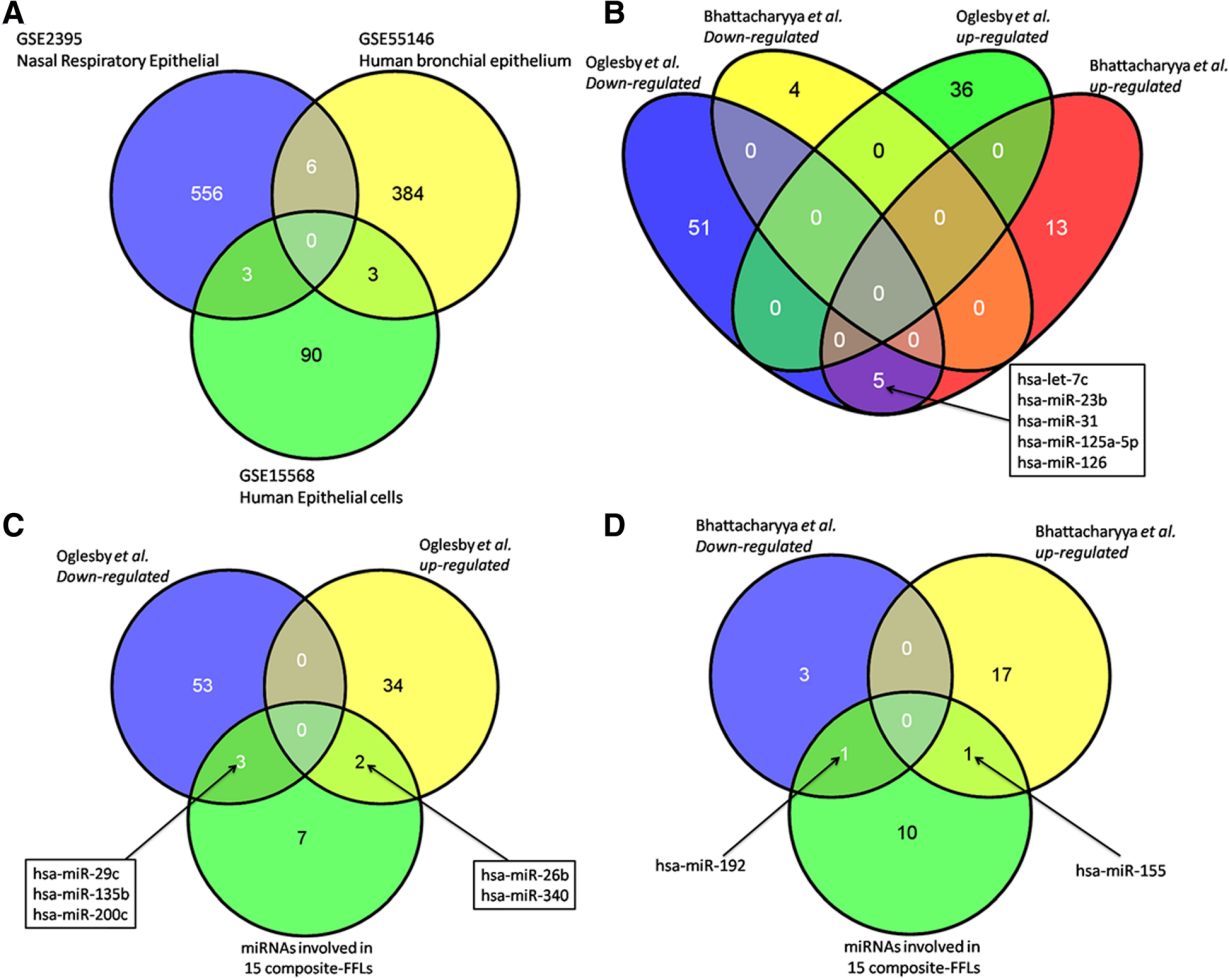
**VENN diagram of CFTR- and CF-related gene and miRNA expression changes. (A)** Common genes among three independent CF patient-related whole genome gene expression data sets; **(B)** common miRNAs identified in bronchial brushings from CF patients or CF bronchial epithelial cell lines; **(C)** commonality among 112 CFTR- and CF-related miRNAs derived from the study of [40]; **(D)** commonality among 112 CFTR- and CF-related miRNAs derived from the study [41].

For this purpose, the publically available GEO data sets GSE2395, GSE55146, and GSE15568 were analyzed. The data informed on whole genome gene expression profiling in cystic fibrosis patients with mild and severe lung disease using either tissue samples obtained from bronchial brushings or nasal epithelium as well as rectal epithelia of CF and non-CF individuals. In all 1,042 DEGs were obtained, however there was little to no overlap among DEGs when individual studies were compared (see Figure 2A).

Furthermore, to discriminate CF-specific and CFTR-related miRNA networks profiling data obtained from CF patient airway epithelium and CF related cell lines as well as primary human airway epithelium were considered using the findings reported by Oglesby *et al.* [40] and Bhattacharyya *et al.* [41]. As denoted for the whole genome gene expression profiling studies major discrepancies among the reported miRNA profiling studies were observed with little overlap in identified miRNAs using either bronchial brushings from CF patients or CF bronchial epithelial cell lines (see Figure 2B). In regards to the CFTR associated miRNA regulations the data reported by Ramachandran *et al.* [25] were used and yielded 112 differential expressed miRNAs. As depicted in Figure 2C, 31 down- and 12 upregulated miRNAs were in common when the findings of Oglesby *et al.* and CFTR-associated miRNAs were compared as determined in human airway epithelium. Likewise, two down- and 10 upregulated miRNAs were commonly regulated when the data reported by Bhattacharyya *et al.* and findings from CFTR-associated miRNAs were compared (Figure 2D). Taken collectively, a total of 93, 22, and 112 uniquely regulated miRNAs were extracted from experimental data and among the three studies seven miRNAs were in common that permitted an in-depth assessment of the miRNA-CF disease relationship.

Apart from CF-specific gene and miRNA expression changes several of the identified genes are also co-expressed or are involved in the same pathways or biological process as determined for the CFTR-associated gene network using human airway epithelium. This is consistent with our understanding of the pathogenesis of CF with most of the common regulated genes influencing folding, sorting, and degradation of proteins and included the ubiquitin mediated proteolysis, protein processing in the endoplasmic reticulum and the proteasome. It has been established that the ubiquitin-proteasome pathway controls the degradation of CFTR and therefore plays a central role in CF .

To be able to construct FFLs, different types of regulatory relationships were considered, that is, genes regulated by either miRNA (miRNA → gene) or TF (TF → gene), as well as the relationships between miRNA regulating TFs and vice versa (miRNA → TF and TF → miRNA) in addition to the gene-gene interaction as depicted in the work flow diagram (Figure 1 and Table 2). The findings entrained on the CFTR gene and miRNA networks were validated using data derived from CF patients as detailed in Table 2.

**Table 2:** **Summary of five different kinds of regulatory relationship and constructed FFLs**

**Relationship**		**Counts**	**miRNAs (n)**	**mRNAs (n)**	**TFs (n)**
Five regulatory relationships	miRNA → mRNA	1,615	99	226	-
	miRNA → TF	422	89	-	52
	TF → miRNA	3,295	102	-	114
	TF → mRNA	16,860	-	387	105
	gene-gene	237		123	
Three categories of FFLs	miRNA FFLs	41	27	128	26
	TF FFLs	393	74	218	64
	Composite FFLs	15	12	104	11

### miRNA gene target relationship

Initially, the miRNA targets were predicted using TargetScan (see the Method Section for further details and Table 2). There were a total of 1,615 miRNA → gene pairs, which involved 99 CFTR specific miRNAs (out of 112 miRNAs identified) and 226 CFTR-regulated genes (out of 419 genes identified from Reference [25]). Among them, the miRNAs, *hsa-miR-200b*, *hsa-miR-200c*, and *hsa-miR-429* regulated the largest number of genes. The average number of targeted genes per miRNA is 16.

It is well known that the miRNAs from the same family share similar regulatory functions and mechanisms [63]. We therefore constructed a miRNA-based network using the CF gene information and investigated whether the relationship between the miRNAs from the same family was preserved as a means to verify the chosen approach. Figure 2 depicts the miRNAs network module where each node is a CF miRNA while an edge denotes the Tanimoto similarity between each of the two miRNAs. It can therefore be demonstrated that the miRNAs from the same family (for example, *hsa-let-7a/b/c/e/g*) were preserved with higher Tanimoto similarity. Likewise, in the constructed miRNA-gene network *NEDD4L* was regulated by 33 miRNAs. This gene codes for an E3 ubiquitin protein ligase and knockdown of *NEDD4L* in lung epithelia causes airway mucus obstruction, goblet cell hyperplasia, inflammation, fibrosis, and even death after 3 weeks of exposure in an animal disease model [64]. Such experimental data support the relevance of the constructed miRNA-gene network.

Using ChIPBase, a total of 422 miRNA → TF pairs were identified and consisted of 89 CFTR-specific miRNAs and 52 human TFs. Among the 422 miRNA → TF pairs, hsa-miR-27a was involved in the regulation of 14 TFs. Meanwhile, the genes *BCL11A*, *SMAD2/3*, and *SMAD4* were regulated by the largest number (n =30) of miRNAs. Note, reduced *SMAD3* protein expression and altered TGFβ1-mediated signaling in CF epithelial cells were reported [65].

### TF-miRNA/gene regulatory networks

TF → miRNA circuitries were constructed using information retrieved from ChIPBase [46]. A total of 3,295 TF → miRNA combinations were computed and this involved 114 and 102 unique TFs and miRNAs, respectively (see Additional file 3: Table S3). For instance, hsa-miR-106b, hsa-miR-25, and hsa-miR-93 were regulated by 72 TFs. Similarly, a total of 16,860 TF-gene pairs were computed and involved 105 TFs and 387 gene targets (see Additional file 3: Table S3). Of the 99 TFs, c-Myc targeted the largest number of CFTR-related genes. It was earlier demonstrated that proteolysis of c-Myc *in vivo* is mediated by the ubiquitin-proteasome pathway [66]. Among the 387 CFTR-related genes, the gene regulated by the largest number of TFs was UBE2D3 (ubiquitin-conjugating enzyme E2 D3). We further searched for common genes among CFTR and 1,042 DEGs and found 38 genes to be mutual.

### CFTR-specific feed-forward loops (FFLs)

It had been demonstrated that composite FFLs (that is, the combined miRNA and TF participating in the regulation of target genes) are more effective in unveiling disease mechanisms than single one as denoted by TF → miRNA or miRNA → TF considerations [45]. As shown in the third step of Figure 1 and as summarized in Table 2, FFLs were evaluated for their significance using a hypergeometric test with multiple testing corrections. Such analysis revealed 449 unique CFTR-entrained FFLs including 41 miRNA-FFLs, 393 TF-FFLs, and 15 composite-FFLs, as shown in Additional file 3: Table S3. The results indicated that the constructed composite-FFLs were of largest relevance followed by miRNA-FFLs and TF-FFLs. Therefore, and based on statistical significance the 15 composite FFLs were employed to search for repurposing candidates for the treatment of CF (Additional file 4: Figure S1). These FFLs contained 12 miRNAs, 11 TFs, and 104 CFTR-related genes, respectively.

We further considered the results of the Kyoto Encyclopedia of Genes and Genomes (KEGG) pathway analysis for the commonly targeted genes of the 15 composite-FFLs. Some composites FFLs, such as *hsa-miR-192*↔CTCF and *hsa-miR-191*↔TCF7L2, just have one gene in common; thus no enriched pathways were obtained. As depicted in Additional file 5: Figure S2, 24 different pathways belonging to 13 different functional categories were considered. Among them, two pathways, ubiquitin-mediated proteolysis and protein processing in the endoplasmic reticulum, dominated the composite FFLs, whose major function is folding, sorting, and degradation and these are key mechanism in CF [67]. Other FFLs are involved in insulin and TGF-beta signaling pathways and endocytosis. For instance, CF-related diabetes (CFRD) is a common complication of CF and insulin resistance may also affect lung function [68]. Likewise, transforming growth factor-beta (TGF-beta) plays a central role in fibrosis, contributing to the influx and activation of inflammatory cells, the epithelial to mesenchymal transdifferentiation (EMT) of cells, and the activation of fibroblasts and modulation of extracellular matrix production [69]. Downregulation of CFTR by TGF-beta limits epithelial chloride secretion, which causes mucus block [70]. It was also reported that CF is associated with a defect in apical receptor-mediated endocytosis [71].

### Validation of FFLs in CF patient samples

To determine disease relevance of the FFLs and to study protein-protein-interactions (PPI) among members of the composite FFLs the following data were considered: (1) whole genome gene expression data; and (2) miRNA profiling studies using samples obtained from bronchial brushings or nasal epithelium as well as rectal epithelia of CF patients with mild and severe disease and non-CF individuals.

**Figure 3:**
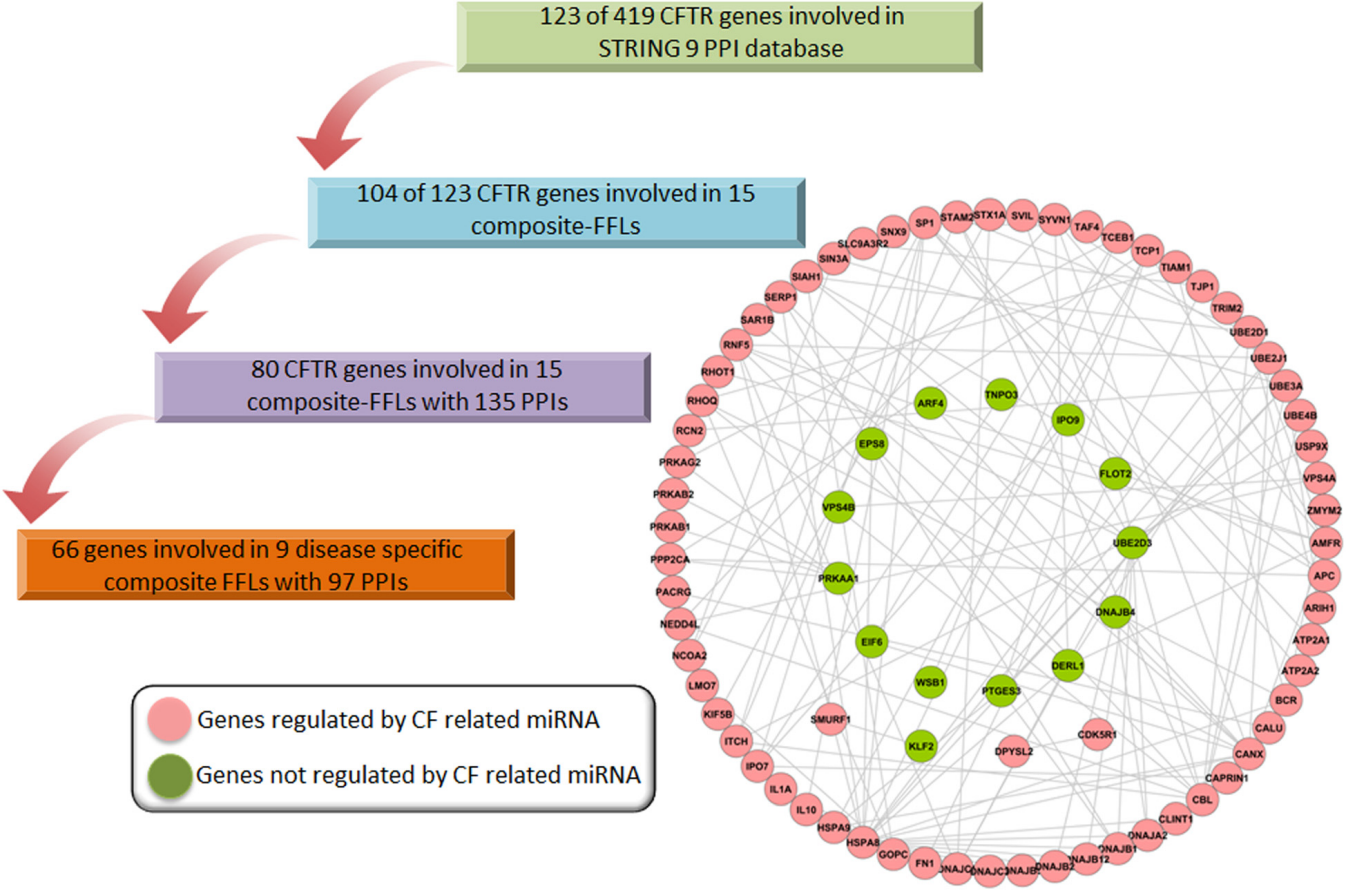
**Protein-protein interaction networks of CFTR-related genes.** A total of 419 genes were retrieved from the study of Ramachandran *et al.* of which 123 could be mapped to the STRING database version 9.1. Only PPI interaction for *homo sapiens* were considered and a confidence score >0.4 was requested. Among them we considered those genes linked to the 15 composite FFL that were constructed. This revealed 80 genes and a total of 135 PPIs to be in common. Subsequently, for nine disease-specific composite FFLs 66 genes and 97 PPIs were observed.

Initially, a total of 123 CFTR genes were retrieved from the study of Ramachandran *et al.* and for 80 genes a total of 135 PPIs were observed in STRING network analysis. This demonstrates that the network partners actually interact with each other. Moreover, for seven and nine disease-regulated miRNAs and TFs, respectively, a total of 97 PPIs among 66 regulated genes were observed further evidencing interactions among the predicted targets (see Figure 3).

**Figure 4:**
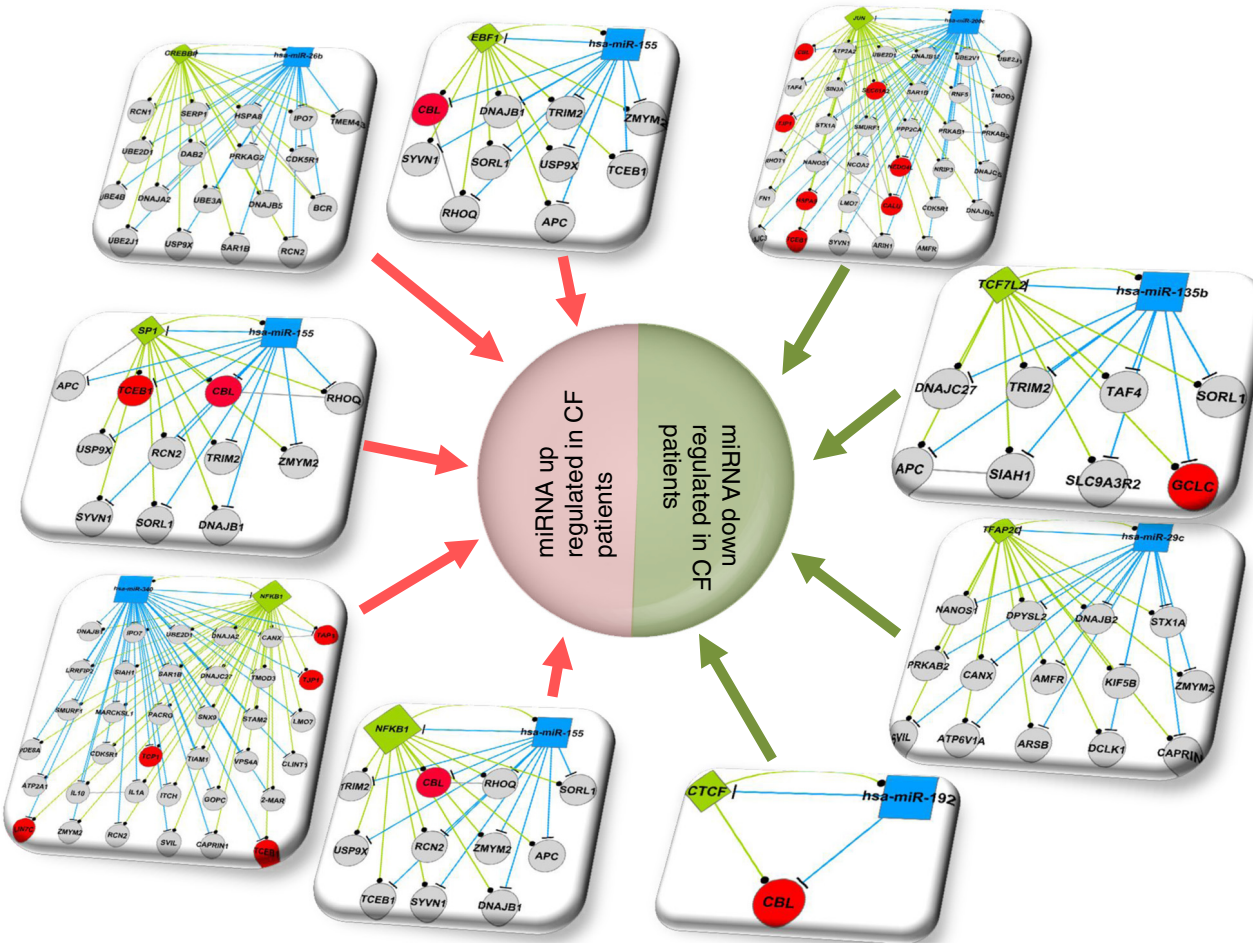
**Composite FFLs of CF-regulated miRNAs.** The nodes are marked as green diamonds whereas blue rectangles and gray ellipses denote transcription factors (TF), miRNAs and genes, respectively. Genes color-coded in red are among the 1,042 CF gene expression data retrieved from three independent CF gene expression data sets. The edges are t-shapes, circle-shapes, and gray solid lines, which denotes miRNAs regulating genes/TF and TFs regulating genes/miRNAs, and gene-gene interaction, respectively.

**Figure 5:**
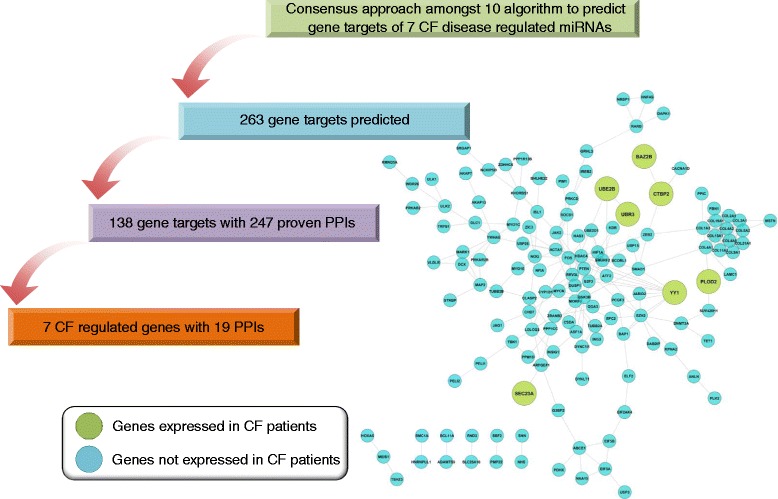
**Protein-protein interaction networks of CF-related miRNA.** A total of 7 CF regulated miRNAs were used to predict gene targets by employing a total of 10 different algorithms. This defined 263 putative targets which were mapped to the STRING database version 9.1 and revealed 247 PPI among 138 gene targets. Only PPI interaction for *homo sapiens* were considered and a confidence score >0.4 was requested. The predicted 138 gene targets were mapped to 1,042 DEGs identified among three independent CF patient-related whole genome gene expression data sets. This identified seven genes in common and a total of 19 PPI.

Subsequently, we considered disease regulated miRNA and its directionality based on CF patient samples and therefore analyzed the data of Oglesby *et al.* [40] and Bhattacharyya *et al.* [41] with respect to the composite FFLs. This revealed a total of seven miRNAs (hsa-miR-26b, hsa-miR-29c, hsa-miR-135b, hsa-miR-155, hsa-miR-192, hsa-miR-200c, and hsa-miR-340) and nine FFLs to be CF associated. Note, in the case of miR-155 three different TFs are involved, that is, SP1, NFKB1, and EBF1, therefore giving rise to three distinct disease-relevant FFLs. We considered miRNAs whose expression was either increased or decreased in CF patient samples (see Figure 4). In order to predict targets of disease associated FFLs we employed a consensus approach using 10 different algorithms (see Additional file 6: Figure S3). The predicted gene targets were considered positive only when confirmed by at least eight different algorithms. Apart from disease specific miRNAs that were used to construct FFLs the regulation of target genes was also considered in CF patient samples. As described above we compiled a total of 1,042 DEGs derived from GEO submissions GSE2395, GSE55146, and GSE15568, and observed DEGs to be commonly regulated in CF samples and disease-specific FFLs, once again providing evidence for the clinical relevance (see Figure 5)

.

### Repurposing candidates for the treatment of CF

Drug repositioning is a process of identifying alternative indications for existing drugs with acceptable safety at affordable price. To identify drugs with potential use in the treatment of CF patients, we exploited small molecules that affect the expression of miRNA which are part of the composite FFLs. We retrieved data from two databases (that is, SM2miR and Pharmaco-miR) as described in Figure 1. Notably, the SM2miR compiles a list of small molecules that interact with miRNAs from the literatures while Pharmaco-miR provides the drug-miRNA association based on the PharmGKB data [72]. We then compared the marketed drug list from DrugBank (version 3.0, [73]) with those identified by SM2miR and Pharmaco-miR as having an ability to influence the expression of miRNA which are part of the FFLs. This process identified 48 unique drugs being strongly designated as repurposing candidates for the treatment of CF patients.

To assess the validity of the CF repurposing candidates, we conducted a two-step analysis. First, we queried clinicaltrials.gov (www.clinicaltrials.gov) that archives clinical studies of human subjects conducted around the world. Collectively, Table 3 compiles all 48 repurposing candidates for CF along with their original indications and literature/clinical trial data. Note that eight out of 48 drugs were already investigated for the treatment of CF patients. For the remaining drug candidates we additionally queried PubMed using the keyword (‘drug name’ (and) ‘cystic fibrosis’) followed by reading. Here, 18 out of 43 repurposing candidates have literature citations to support their potential use in CF. Collectively, we found 54.2% of the candidates (26 drugs out of 48 repurposing candidates) to have at least one published study or clinical trial related to CF. Additional file 7: Table S4 lists the information of all 48 repurposing candidates related to drug safety and affordability that were obtained from the FDA-approved drug product labels and the DrugBank V3.0 database.

**Table 3:** **Summary information of 48 repurposing candidates for cystic fibrosis (CF) treatment**

**Repurposing candidates**	**Original indications**	**Notes**	**Confirmation sources**
***Evidence found from clinic trials (clinicaltrials.gov)***			
Simvastatin	Hypercholesterolemia; lower cholesterol	Simvastatin will increase nitric oxide (NO) produced (exhaled NO), and may decrease airway inflammation	NCT00255242
Pyruvic acid	Dietary shortage or imbalance	The inhalation of sodium pyruvate may reduce lung damage in patients with CF by its ability to reduce levels of toxic reactive oxygen and nitrogen compounds associated with the chronic inflammatory component of the disease	NCT00308243
Pioglitazone	Type 2 diabetes	Pioglitazone may decrease inflammation in CF lung disease	NCT00322868
l-Glutamine	Dietary shortage or imbalance	Patients with CF develop frequent and potentially life-threatening lung infections. The nutrient glutamine may help the body fight off infection	NCT01051999
Desipramine	Neuropathic pain; attention deficit hyperactivity disorder; anxiety disorders	Combination of desipramine and VX-770 for CF treatment	NCT01153542
Nitric oxide	Neonates with hypoxic respiratory failure	Exhaled nitric oxide (NO), elevated in most inflammatory lung diseases, is decreased in CF, suggesting decreased formation, increased metabolism or loss of NO	NCT00570349
			PMID: 15982933
Choline	Dietary shortage or imbalance	Nutrition and methyl status of children with CF could be improved after supplying a choline supplement	NCT01070446
l-Leucine	Prevention of the breakdown of muscle proteins	A high-leucine essential amino acids mixture specifically designed to stimulate protein anabolism could target the metabolic alterations of pediatric subjects with CF	NCT01172301
***Literature support***			
Fluoxetine	Depression; obsessive-compulsive disorder; antiviral	It was suggested that a hydrophobic interaction with high affinity between uncharged fluoxetine and volume-activated chloride channels. Ca^2+^-activated Cl^-^ currents and CFTR are also blocked by fluoxetine, revealing a novel characteristic of the drug as a chloride channel modulator	PMID: 10077245
Cyclosporine	Transplant rejection; rheumatoid arthritis; severe psoriasis	These results suggest that cyclosporine can be beneficial as a steroid sparing agent in CF patients	PMID: 11213776
Morphine	Severe pain	Inhaled morphine to relieve dyspnea in patients with end-stage lung disease due to CF	PMID: 10973044
Methotrexate	Gestational choriocarcinoma; chorioadenoma destruens; hydatidiform mole; psoriasis; rheumatoid arthritis	It was suggested an effective systemic anti-inflammatory effect of methotrexate in treatment for CF patients with advanced pulmonary disease	PMID: 12735666
Vitamin C	Used to treat vitamin C deficiency, scurvy, delayed wound and bone healing, urine acidification, and in general as an antioxidant. It has also been suggested to be an effective antiviral agent	The pool of vitamin C in the respiratory tract represents a potential nutraceutical and pharmaceutical target for the complementary treatment of sticky airway secretions by enhancing epithelial fluid secretion	PMID: 14993613
Dexamethasone	Anti-inflammatory; oncologic uses; glucocorticoid resistance; obstetrics; high altitude illnesses	Low doses of dexamethasone constantly delivered by autologous erythrocytes slow the progression of lung disease in CF patients	PMID: 15223012
l-Arginine	Treating dietary shortage or imbalance	It was suggested that airway nitric oxide formation in CF patients can be augmented with oral L-arginine supplementation	PMID: 15640324
Tacrolimus	Atopic dermatitis; organ rejection	Tacrolimus was tested on lung transplantation of CF patients	PMID: 16372829
Tamoxifen	Breast cancer	This inhibition of Ca^2+^ signaling was prevented and even potentiated by estrogen antagonists such as tamoxifen, suggesting that antiestrogens may be beneficial in the treatment of CF lung disease because they increase Cl^-^ secretion in the airways	PMID: 19033671
Rosiglitazone	Type 2 diabetes	It was suggested rosiglitazone as important modulators of intestinal Cl- secretory function	PMID: 19443733
Vorinostat	Cutaneous T cell lymphoma	Vorinostat (SAHA) could restore surface channel activity in human primary airway epithelia to levels that are 28% of those of wild-type CFTR	PMID: 19966789
Metformin	Type 2 diabetes; prediabetes; polycystic ovary syndrome; gestational diabetes	The metabolic sensor AMP-activated kinase (AMPK) inhibits both the CFTR Cl(-) channel and epithelial Na(+) channel (ENaC), and may inhibit secretion of proinflammatory cytokines in epithelia	PMID: 19617399
Estradiol	Urogenital	17Beta-estradiol inhibits IL-8 release by ERbeta in CF bronchial epithelial cells through upregulation of secretory leucoprotease inhibitor, inhibition of nuclear factor (NF)-kappaB, and IL-8 gene expression. These data implicate a novel anti-inflammatory mechanism for E(2) in females with CF, which predisposes to infection and colonization	PMID: 20378727
Chloroquine	Malaria; strains of *P. falciparum*; rheumatoid arthritis	Vasculitis is a well recognized complication of CF. There is a case of steroid-resistant cutaneous vasculitis which was successfully treated with chloroquine in addition to corticosteroids and a subsequent relapse with chloroquine alone	PMID: 20863769
Sirolimus	Prophylaxis	Autophagy stimulation by sirolimus (rapamycin) suppresses lung inflammation and infection by Burkholderia cenocepacia in a model of CF	PMID: 21997369
Nifedipine	Vasospastic angina; chronic stable angina; hypertension; Raynaud’s phenomenon	Nifedipine may be a useful adjuvant to supplemental oxygen in the treatment of patients with CF and cor pulmonale	PMID: 6476600
Levamisole	Dukes’ stage C colon cancer; worm infestations	Levamisole could block K+ channels required for Cl(-)-secretory responses elicited by diverse pathways in model epithelia and native colon, an effect that outweighs their ability to activate apical Cl- channels	PMID: 9609763
Adenosine triphosphate	Dietary shortage or imbalance	It was reported that extracellular adenosine triphosphate (ATP) and adenosine were important luminal autocrine and paracrine signals that regulated the hydration of the surface of human airway epithelial cultures through their action on apical membrane purinoceptors	PMID: 23757023
***Potential candidates for CF treatment***			
Imiquimod	non-hyperkeratotic; non-hypertrophic actinic keratoses		
Phenobarbital	Seizures		
Leucovorin	Osteosarcoma		
Imatinib	Philadelphia chromosome positive chronic myeloid leukemia (CML); malignant gastrointestinal stromal tumors (GIST)		
Gemcitabine	Metastatic breast cancer		
Dienestrol	Atrophic vaginitis; kraurosis vulvae		
Cyclophosphamide	Malignant lymphomas; multiple myeloma; leukemias		
Chlorotrianisene	Menopause		
Amiodarone	Cardiac dysrhythmias		
Warfarin	Retinal vascular occlusion; pulmonary embolism; cardiomyopathy; atrial fibrillation and flutter; cerebral embolism; transient cerebral ischemia; arterial embolism; thrombosis		
Paclitaxel	Kaposi’s sarcoma; cancer of the lung; ovarian and breast		
Oxaliplatin	Cancer chemotherapy		
Imipramine	Depression		
Fluorouracil	Multiple actinic; solar keratoses		
Mercaptopurine	Acute lymphatic leukemia		
Cetuximab	EGFR-expressing metastatic colorectal cancer; squamous cell carcinoma		
Dopamine	Hemodynamic imbalances		
Vinblastine	Breast cancer; Hodgkin’s and non-Hodgkin’s lymphomas; Kaposi’s sarcoma		
Adenosine monophosphate	Dietary shortage or imbalance		

**Figure 6:**
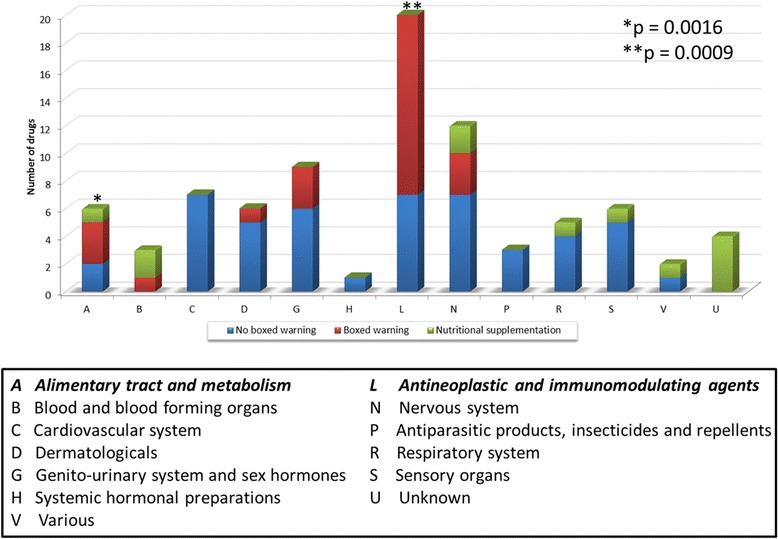
**The distribution of repurposing candidates for CF at the first level of Anatomical Therapeutic Chemical Classification System (ATC).** Each bar was divided by safety concerns including boxed warning, no boxed warning, and nutritional supplementation. The statistical significance of the therapeutic categories associated with CF are A and L based on the Fisher’s exact test with a *P* value cutoff of 0.01.

We further assessed the therapeutic indications of the repurposing candidates and found two categories, that is, Alimentary tract and metabolism (*P* <0.0016) and Antineoplastic and immunomodulating agents (*P* <0.0009) to be significantly enriched. For the different therapeutic categories of the 48 drug repurposing candidates see Figure 6. Note that the two therapeutic categories include some drugs with boxed warning that need to be considered.

## Discussions

This study aimed to define suitable drug repurposing candidates for the treatment of CF. For this purpose, FFLs were entrained on CFTR and CF gene networks. Applying FFLs to an entire drug landscape is a complex undertaking and next to safety, affordability was considered. In all 41 miRNA-FFLs, 393 TF-FFLs, and 15 composite FFLs were computed. Using diverse computational strategies gene targets were predicted based on disease-regulated miRNA and involved a consensus approach among different algorithms (see Additional file 6: Figure S3). Validation was achieved with CF patient sample-specific information and FFLs were used to enrich the repurposing drug candidate pipeline by considering small molecules effects on miRNA expression. Eventually, 48 repurposing candidates were obtained; their usefulness was considered based on clinical trial information, literature findings, safety concerns, and affordability points of view.

Based on its ability to influence *miR-26b* [74] and the transcription factor *CREBBP* [75], dexamethasone was considered as a repurposing candidate. Dexamethasone is a potent steroid and acts as an anti-inflammatory and immunosuppressant. Its use in CF patients is consistent with the current practice of glucocorticoids in the treatment of lung inflammation [76]. It was reported that low doses of dexamethasone delivered by autologous erythrocytes slows the progression of lung disease in CF patients [77]. As dexamethasone is an approved prescription drug without boxed warning, it provides additional value for its application in CF.

The employed testing strategy also predicted statins as interesting repurposing candidate and it was reported that statins retain ceramide levels normal in CF patients [78,79]. As ceramides and sphingolipids are components of lipid rafts, they play deceive roles in transmembrane signaling [80]. We found simvastatin to be implicated in the *hsa-miR-200c*↔*JUN* regulatory FFL. However, some statins are associated with severe adverse drug reactions, most notable rhabdomyolysis. For this reason cerivastatin had been withdrawn from the market [81]. In a clinical trial (NCT00255242) the beneficial effect of simvastatin in alleviating airway inflammation in CF patients was investigated [82] and statins without boxed warning in the label are interesting repurposing candidates.

Likewise, phenylimidazothiazoles were reported to activate wild-type and mutant CFTR in transfected cells and thus, have been proposed as drug remedy for CF [83,84]. The present study inferred levamisole to perturb the *hsa-mir-26b*↔*CREBBP* and *hsa-miR-200c*↔*JUN* FFLs and this drug was used to treat Dukes’ stage C colon cancer and worm infestations. It was demonstrated that levamisole inhibited intestinal Cl^-^ transport *via* basolateral K^+^ channel blockade [85] and this provides a molecular rational for further evaluation.

In another clinical trial (NCT01070446), the effects of choline supplementation in children with CF was investigated. Note, children with CF are reported to have depleted levels of choline [86] and choline is involved in two composite FFLs including *hsa-miR-200c*↔*JUN* and *has-miR-29c*↔*TFAP2C* as per our investigations.

Furthermore, among the repositioning candidates were thiazolidinediones (TZD), which were initially used to treat type-II diabetes. This class of drugs includes both pioglitazone and rosiglitazone. Some evidence exists that TZD could be used to ameliorate the severity of the CF phenotype [87]. Pioglitazone and rosiglitazone are known to activate peroxisome proliferator-activated receptor gamma (PPARγ) and it has been suggested that a reversible defect in PPARγ signaling in *Cftr*-deficient cells could improve the severity of the CF phenotype in mice. Additionally, in clinical trial NCT00322868 pioglitazone was evaluated for its ability to decrease inflammation in CF lung patients. While thiazolidinediones are promising, unfortunately this class of drug has been reported to exacerbate congestive heart failure in some patients and thus are labelled with a boxed warning. In addition, troglitazone was withdrawn from the market due to severe liver toxicity [88].

The performed analysis also identified some anti-cancer drugs for potential use in CF patients [89,90] and it was hypothesized that induction of homologous recombination in respiratory epithelium helps to improve the lung function of patients [90]. In our analysis, vorinostat (SAHA), a histone-deacetylase inhibitor and anti-cancer drug used to treat cutaneous T-cell lymphoma, appeared to be a reasonable repurposing candidate. This drug was reported to restore surface channel activity in human primary airway epithelia to a level that was 28% of wild-type CFTR and does not have a boxed warning, but is fairly expensive.

The developed drug repurposing strategy is a modular system and each of the components can be modified or even replaced by other algorithms. Besides TargetScan there are alternatives approaches such as RNAhybrid [56], DIANA-microT [52], RNA22 [59], miRanda [53], PicTar [57], and miRWalk [55] to predict gene targets. Indeed, it has been suggested that combining diverse algorithms could provide more confidence in the inferred drug miRNA-gene relationships [20,91]. To this effect we employed a consensus approach for predicting gene targets of disease-regulated miRNAs by using 10 different algorithms. Lastly, the directions of the disease-regulated miRNAs were considered and the consensus approach revealed that 48% of the predicted targets by TargetScan could be confirmed by at least another algorithm. Moreover, for the TF binding site predictions, we utilized experimental data retrieved from ChIP-Seq experiments. Other technologies such as CHIP on chip array or *in silico* approaches based on position weight matrices can also be employed to identify putative transcription factor binding sites [92].

The present study focused on repurposing candidates from the miRNA-small molecule perspective while the relationship between TFs and small molecules, that is, drugs affecting transcription factor expression and activity was not considered in detail. So far only a few drugs target TF and would therefore limit the choice of drug repurposing candidates; nonetheless drugs targeting TFs will increase with time [93]. A recent study demonstrated heat shock transcription factor 1 (*HSF1*) as a potential new therapeutic target in multiple myeloma [94] and other studies revealed that nuclear transcription factor-kappa B (*NFκB*) could be a potential target for drug development in different disease entities [95-97]. We also found *NFκB* to be involved in several composite-FFLs including *has-miR-155↔NFκB* and *has-miR-340↔NFκB*. Note that ibuprofen, a non-steroidal anti-inflammatory drug (NSAID), is one of the anti-inflammatory therapies used in the treatment of CF [98,99]. It has been demonstrated that high dose ibuprofen causes modest suppression of *NFκB* transcriptional activity in CF respiratory epithelial cells. Furthermore, *miR-155* promotes inflammation in CF by driving hyper-expression of interleukin-8 [41]. In future studies, the transcription factor drug relationship will be explored in greater detail.

The goal of drug repurposing is to bring new therapies to the market at a lower risk, reduced cost, and less development time when compared to conventional drug development programs [100]. However, the safety assessment in a new disease indication is still an important concern in the regulatory process. While the safety assessment is based on drug label information, the drug repositioning approach may involve different formulations and changes in dosage that need to be considered in different patient populations. For instance, the use of high dose ibuprofen in CF is concerning for its adverse drug reactions, most notably in causing GI hemorrhage, myocardial infarction, drug-induced liver injury, and even renal failure [7]. Additional approaches for safety assessment of market drugs are the U.S. FDA Adverse Event Reporting System (FAERS) [101] and the FDA’s Sentinel initiative [102]. Finally, 10% of healthcare expenditure in the U.S. has been attributed to prescribed drugs [103]. Thus, drug affordability will require consideration.

## Conclusion

In conclusion, we report a strategy for the rational selection of drug repurposing candidates based on miRNA-TF FFLs. The methodology developed is straightforward and may also apply for the selection of drug candidates in other rare diseases.

## Additional files

Additional file 1: Table S1. Cystic fibrosis related genes from diverse of resources. Additional file 2: Table S2. CFTR associated miRNAs. Additional file 3: Table S3. The enriched cystic fibrosis related feed forward loops (FFLs). Additional file 4: Figure S1. The composite feed-forward loops (FFLs) for CF. The nodes are green diamonds, blue rectangles, and gray ellipses which denote transcription factors (TFs), miRNAs, and genes, respectively. The edges are t-shapes, circle-shapes, and gray solid lines, which represent repression of miRNAs to genes/TF, regulation of TFs to genes/miRNAs, and gene-gene interaction, respectively. Additional file 5: Figure S2. Network of composite-FFLs and enriched KEGG pathway based on the common targeted genes of the composite-FFLs. The KEGG pathways are tested for statistical significance by using the Fisher’s exact test with multiple testing corrections. Only the pathways with corrected *P* values less than 0.05 were considered enriched. Additional file 6: Figure S3. Comparsion of miRNA target predictions for miRNAs involved in 15 composite FFLs based on 10 different algorithms. Additional file 7: Table S4. The information of 48 enriched candidates for cystic fibrosis.
