# Supplementary figures and images for: Deciphering miRNA transcription factor feed-forward loops to identify drug repurposing candidates for cystic fibrosis
Source: Genome Med. 2014 Dec 2;6(12):94. doi: 10.1186/s13073-014-0094-2 (PMC4256829; doi:10.1186/s13073-014-0094-2)

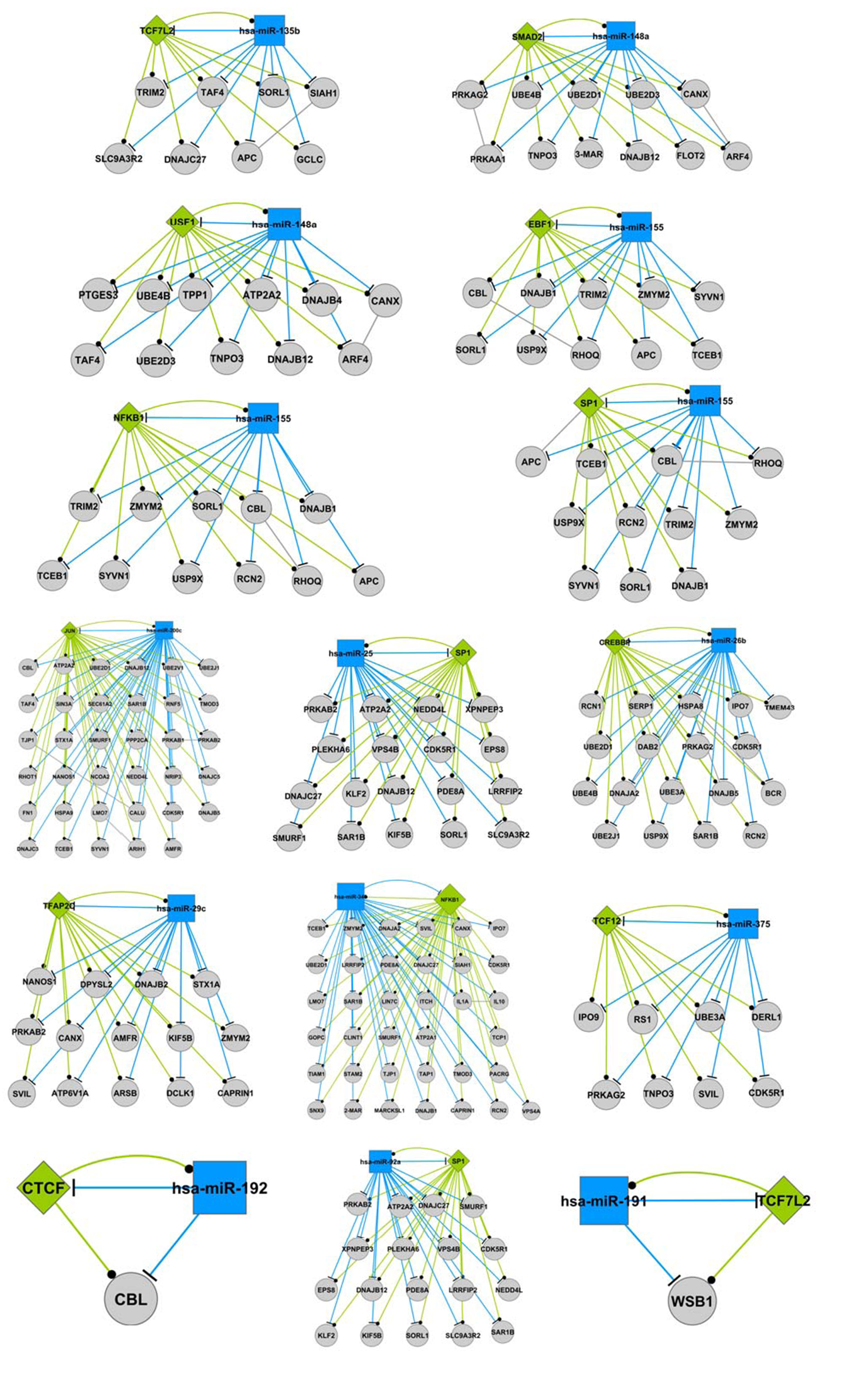

Supplement: Additional file 4: Figure S1. — The composite feed-forward loops (FFLs) for CF. The nodes are green diamonds, blue rectangles, and gray ellipses which denote transcription factors (TFs), miRNAs, and genes, respectively. The edges are t-shapes, circle-shapes, and gray solid lines, which represent repression of miRNAs to genes/TF, regulation of TFs to genes/miRNAs, and gene-gene interaction, respectively. [file 13073_2014_94_MOESM4_ESM.tiff]

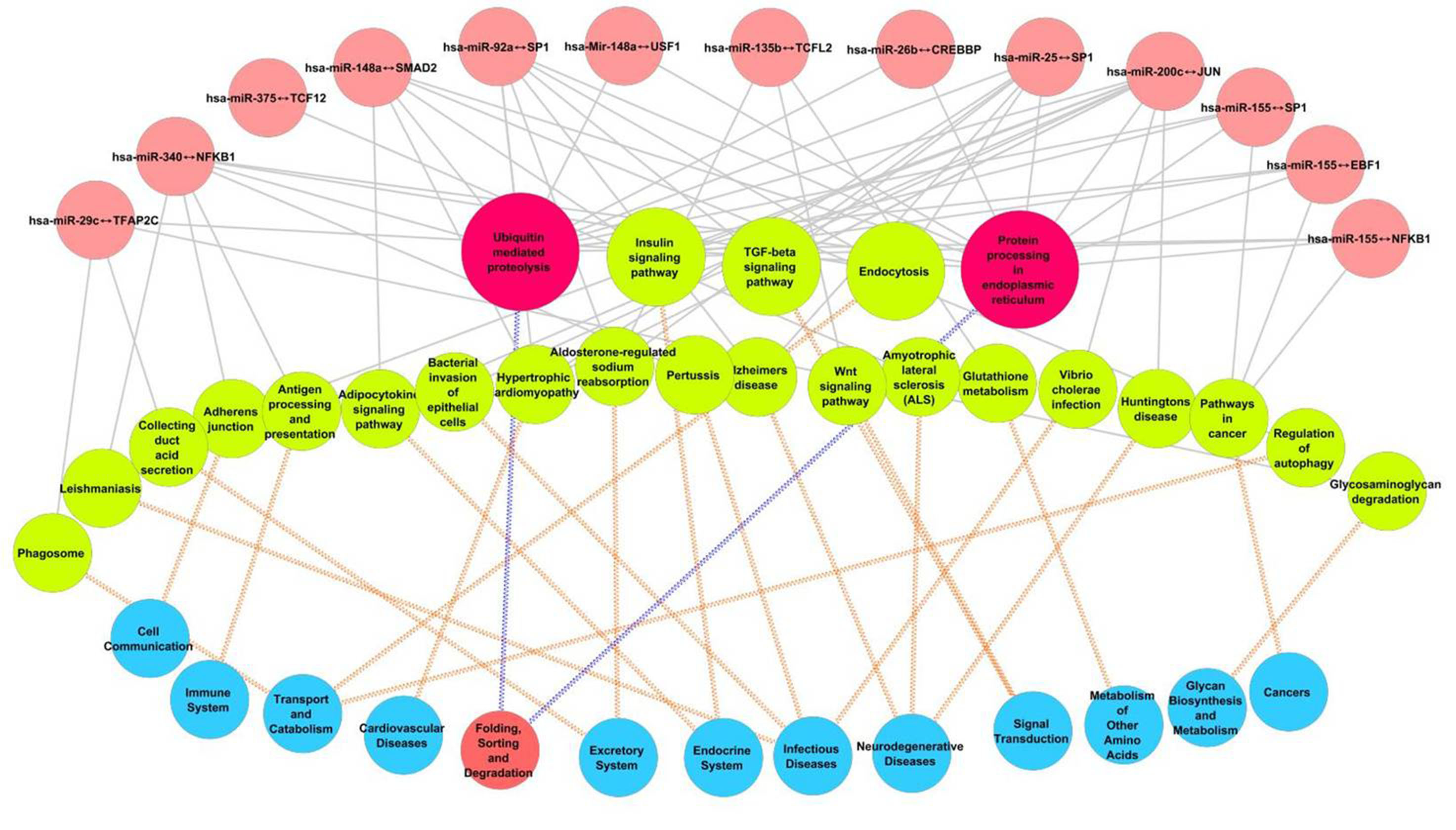

Supplement: Additional file 5: Figure S2. — Network of composite-FFLs and enriched KEGG pathway based on the common targeted genes of the composite-FFLs. The KEGG pathways are tested for statistical significance by using the Fisher’s exact test with multiple testing corrections. Only the pathways with corrected P values less than 0.05 were considered enriched. [file 13073_2014_94_MOESM5_ESM.jpeg]
